# TNF-α and Temporal Changes in Sleep Architecture in Mice Exposed to Sleep Fragmentation

**DOI:** 10.1371/journal.pone.0045610

**Published:** 2012-09-21

**Authors:** Navita Kaushal, Vijay Ramesh, David Gozal

**Affiliations:** Department of Pediatrics, Section of Pediatric Sleep Medicine, The University of Chicago, Chicago, Illinois, United States of America; Pennsylvania State University, United States of America

## Abstract

TNF-α plays critical roles in host-defense, sleep-wake regulation, and the pathogenesis of various disorders. Increases in the concentration of circulating TNF-α after either sleep deprivation or sleep fragmentation (SF) appear to underlie excessive daytime sleepiness in patients with sleep apnea (OSA). Following baseline recordings, mice were subjected to 15 days of SF (daily for 12 h/day from 07.00 h to 19.00 h), and sleep parameters were recorded on days1, 7 and 15. Sleep architecture and sleep propensity were assessed in both C57BL/6J and in TNF-α double receptor KO mice (TNFR KO). To further confirm the role of TNF-α, we also assessed the effect of treatment with a TNF- α neutralizing antibody in C57BL/6J mice. SF was not associated with major changes in global sleep architecture in C57BL/6J and TNFR KO mice. TNFR KO mice showed higher baseline SWS delta power. Further, following 15 days of SF, mice injected with TNF-α neutralizing antibody and TNFR KO mice showed increased EEG SWS activity. However, SWS latency, indicative of increased propensity to sleep, was only decreased in C57BL/6J, and was unaffected in TNFR KO mice as well as in C57BL/6J mice exposed to SF but treated with TNF-α neutralizing antibody. Taken together, our findings show that the excessive sleepiness incurred by recurrent arousals during sleep may be due to activation of TNF-alpha-dependent inflammatory pathways, despite the presence of preserved sleep duration and global sleep architecture.

## Introduction

Sleep and wakefulness are controlled by a network of brain nuclei that interact in a complex fashion allowing for integration of homeostatic and circadian regulatory networks [1]. Disruption of sleep integrity, either by sleep deprivation (SD) [2] or by sleep fragmentation (SF) provides a useful tool to interfere with the sleep cycle in both humans [3] and rodents [4], [5], [6] and thus potentially enable a better understanding of the functions of sleep in health and disease.

The mechanisms by which SD or SF induce pathological changes ultimately leading to excessive sleepiness (ES), cognitive deficits and obesity remain unclear. However, the levels of specific cytokines underlying the regulation of the inflammatory response may also play a pivotal role in both sleep regulation and induction of ES. Furthermore, although SD has been found to alter immune responses [7], and to lead to increased circulating levels of pro-inflammatory cytokines such as interleukin-6 (IL-6), tumor necrosis factor-alpha (TNF-α), and C-reactive protein (CRP) [8], [9], prolonged SD conditions in humans are unusual. A few studies have demonstrated association of sleep loss with next-day increase in IL-6 and TNF-α [10], [11], [12] and such changes in inflammatory cytokines have been proposed as mediators of pathological or experimentally-induced sleepiness in humans. In contrast, SF is a much more prevalent condition, and yet the role of SF in modulating pro-inflammatory pathways remains virtually unexplored.

TNF- α is involved in host defense immune responses and in the pathogenesis of various diseases [13], [14]. TNF-α activates a number of inflammatory pathways mainly via NF-κB, a transcription factor that activates nitric oxide synthase, cyclooxygenase 2, and adenosine A1 receptors, which in turn are implicated in sleep regulation [15], [16]. Shoham *et al* in 1987 [17] first reported on the somnogenic properties of TNF-α. In addition TNF-α was also shown to play a major role in the regulation of normal physiologic sleep in the absence of any immune challenge [18]. Indeed, the central or peripheral administration of exogenous pro-inflammatory cytokines such as interleukin 1 and TNF-α will induce increases in NREM sleep in mice [19], rats [20], [21], [22], [23], rabbits [17], cats [24] and monkeys [25], as well as decreases in REM sleep [26]. TNF-α administration will also lead to increases in EEG delta frequency (0.5–4 Hz) power, a popular and widely employed index of sleep intensity [27], [28], [29]. Unilateral application of TNF-α in the cerebral cortex enhances sleep intensity [30], while reduction in TNF-α expression in one brain hemisphere using TNF-α small interfering RNA leads to reduced EEG delta power during NREM only on the affected side of the brain [31]. Similarly, neutralizing endogenous TNF-α antibodies [32] or TNF soluble receptors [23], [33] inhibit spontaneous NREM sleep, and attenuate sleep rebound after sleep deprivation. Levels of TNF-α in brain and plasma in rats are higher during the light period [34] and the highest concentrations of TNF-α mRNA were measured at the onset of the light period in the hypothalamus of rats [35]. In humans, plasma concentrations of TNF-α are circadian phase-dependent and are directly related to delta frequency activity (sleep quality) during sleep [36]. TNF-α plasma levels [37], protein levels and brain expression of TNF-α [16] are enhanced with prolonged wakefulness and the elevated plasma TNF-α levels are associated with enhanced sleep following SD [38].

TNF-α and IL-1β act directly on the neurons of the hypothalamic preoptic area and basal forebrain, the major sleep regulatory areas of the brain [39], [40]. Microinjections of TNF-α in locus ceruleus [20] and anterior hypothalamus [23] enhance NREM sleep in rats. Since pro-inflammatory cytokines interact with neurotransmitters, peptides and hormones, it is postulated that cytokines may mediate sleep through their effects on the somatotropic hormonal system and the hypothalamic pituitary adrenal axis [41].

Two cell surface receptors for TNF-α have been identified, namely 55 kDa or type I receptors and 75 kDa or type II receptors. Since TNF-α 55 kDa receptor knockout mice sleep less than wild type mice and fail to sleep more after exogenous TNF- α administration [19], it is thought that TNF-α affects sleep processes via TNF receptor (TNFR) type I. Although these receptors have distinct *in-vivo* physiological functions in mediating and modulating the biologic activity of TNF-α [42], their mechanism(s) and sites of action in the context of sleep regulation are not completely understood. This issue is particularly important considering that many human diseases linked with sleep disturbances are associated with increased circulating TNF-α levels. For example, patients suffering from insomnia [43], fibromyalgia [44], obesity [45], sleep apnea (OSA) [46], [47], [48], [49] and various infections [50], [51] exhibit elevations in circulating TNF-α levels. Neutralization of TNF-α activity is associated with a significant reduction of objective sleepiness in obese patients with OSA, suggesting that pro-inflammatory cytokines contribute to the pathogenesis of sleepiness in conditions that characteristically exhibit SF as a one of the constitutive elements of the disease [52].

We recently reported that SF for extended periods of time leads to temporally-dependent changes in TNF-α gene and protein expression in brain cortex, and also promotes increased sleep propensity in the absence of compromised total sleep duration [6]. In the present study, we aimed to characterize changes in sleep patterns and sleep propensity in TNF receptor double KO mice (TNFR KO) subjected to SF and further compare these findings to those obtained in wild type mice exposed to SF, but treated with TNF-α neutralizing antibodies.

## Materials and Methods

### Animals

Male C57BL/6J mice (WT) and TNF-α receptor double knockout mice (B6;129S-*Tnfrsf1a^tm1Imx^ Tnfrsf1b^tm1Imx^*/J; TNFR KO) (both weighing 20–25 g) were purchased from Jackson Laboratories, (Bar Harbor, Maine). The mice were housed in a 12 h light/dark cycle (light on 07.00 h to 19.00 h) at a constant temperature (26±1°C) and were allowed access to food and water *ad libitum*. The experimental protocols were approved by the Institutional Animal Use and Care Committee and are in close agreement with the National Institutes of Health *Guide in the Care and Use of Animals*. All efforts were made to minimize animal suffering and to reduce the number of animals used.

### Surgical Procedures

All surgical procedures were performed under sterile conditions and isoflurane general anesthesia: induction, 3% isoflurane and 1 liter per min of O_2_ and maintenance, 2% isoflurane and ½ liter per min of O_2_. First, the animals were positioned in sternal recumbency, and a dorsal neck incision of 2–3 cm was made through the skin along the dorsal midline, covered with a sterile bandage, after which, a 1.5–2 cm incision was performed through the skin and abdominal wall along the ventral midline. A telemetric transmitter weighing 3.5 g, F20-EET (DSI, Minnesota, USA), which allows simultaneous monitoring of two biopotential channels, temperature and locomotor activity was inserted, biopotential leads were exteriorized, and the abdominal wall was closed using 4-0 non-absorbable suture with a simple interrupted pattern. The 2 pairs of biopotential leads were then advanced subcutaneously from the ventral abdomen incision to the dorsal neck incision using a trocar. Animals were then fixed in a stereotaxic apparatus for implantation of EEG electrodes, with the first pair of biopotential leads being fixed to the skull above the frontal area (1 mm anterior to bregma and 2 mm lateral to mid sagittal suture for one of the leads, and 1 mm anterior to lambda and 2.5 mm lateral to mid sagittal suture for the other lead). The other pair of biopotential leads was placed within the same bundle of dorsal neck muscles for the recording of nuchal EMG.

### Acclimatization and sleep recordings

After complete recovery from surgery, mice were transferred to the sleep fragmentation (SF) devices for acclimatization and initial habituation to the sweeper movement. During habituation, the device was switched on for 15 min (2 times per day) at random intervals during the light period. The recording cages were mounted on a DSI telemetry receiver (RPC-1), which were in turn connected to an acquisition computer through a data exchange matrix. After one week of acclimatization in the cages, the magnetic switch of the transmitter was activated, and polygraphic recordings were begun at 7.00 AM. Physiological data were continuously acquired for 24 h using Dataquest ART acquisition software (DSI, Minnesota, USA; version 3.1), at a sampling rate of 500 Hz.

### Sleep Fragmentation

SF was performed by switching on the sweeper to a timer mode in the cage as previously described [5], [6], [53]. In this mode, the sweeper required around 9 sec to sweep the floor of the cage one way. When it reached to the end of the cage, a relay engaged the timer which paused for 2 min before enabling the sweeper to move in the opposite direction. Between the 2 intervals, the animal remained undisturbed. During sweeper motion, animals would need to step over the sweeper, and continue with their unrestrained behavior. If the mouse was asleep, a brief tactile stimulation elicited intermittent brief arousal by the sweeper motion. This method prevents the need for human contact and intervention, and minimizes physical activity during the entire sleep disruption procedure, and closely mimicked the best methodological approach to study sleep disorders such as OSA.

### Sleep-wake parameters

The behavior data were first scored automatically using Sleepsign software (Kissei Comtec, Japan), and records were visually confirmed or corrected as needed. Many researchers have adopted and successfully applied this software for sleep-wake analyses [5], [54], [55]. Behavior was classified into 3 different states: wake, slow wave sleep (SWS) and rapid eye movement (REM) sleep. EEG during W had low-amplitude, high-frequency (desynchronized) waves. During wake, EMG records showed gross body movement artifacts and behaviorally, animals had grooming, scratching and orienting activity. The SWS was characterized by low-frequency, high-amplitude (synchronized) EEG with a considerable reduction in EMG amplitude. The mice assumed a curled recumbent posture during this period. REM sleep was characterized by desynchronized EEG, and a drastic reduction in EMG (muscle atonia). Sleep-related low frequency (delta) activity was also derived from the records using bandpass filtering of 1–4.0 Hz. Delta power was computed by using SleepSign software by Fast Fourier Transform (FFT), which was based on 512 points corresponding to 10 sec epochs, at a sampling rate of 250 Hz with Hanning as the window filter of FFT. The SWS epoch which showed movement artifacts, were excluded when computing delta power, since EEG signals are especially sensitive to movement, with the resulting artifact specifically enhancing signals in the delta band. The mean wake episodes were computed throughout 24 h in 2 h bin. The SWS latency, the time elapsed following a wake episode to the initiation of SWS episode, was calculated for each arousal throughout the 24 h period.

### Group 1: Sleep fragmentation (WT vs. TNFR KO mice)

The SF group included male WT mice (n = 6) and TNFR KO (n = 6) implanted with telemetric transmitters. Baseline recordings were carried out for 24 h from 07.00 h to 07.00 h next day. After the baseline recording was completed, the SF device was turned on and animals were subjected to SF every day for the next 15 days during the light period from 07.00 h to 19.00 h. The sleep recordings were acquired on day 1, day 7 and day 15 of SF.

### Group 2: TNF- α neutralizing antibody injection and sleep fragmentation

Baseline recordings were carried out for 24 h from 07.00 h to 07.00 h next day in male WT mice (n = 12). After the baseline recordings were completed, 6 mice were injected with 100 µg/0.3 ml of TNF-α neutralizing antibody (clone TN3-19.12) i.p., and the other 6 mice were injected with 0.3 ml of sterile normal saline at 07.00 h every morning followed by SF for 15 days. The sleep recordings were acquired on days 1, 7 and 15 of SF.

### Data analysis

In all the experimental conditions, the sleep-wake data were divided into 10 sec epochs and scored. They were then divided into 2-h bins. We used multivariate MANOVA model (SPSS 11, Chicago, IL) to allow full assessment whether different conditions on three different behavioral states were present. The MANOVA model included: 2-hr time bins as within factors (12 time points) and 2 between factors: (1) Condition (four levels): BL, SF day1, SF day7 and SF day 15 (2) State (three levels): wakefulness, SWS, and REM sleep. All F statistics are reported using Pillai's Trace. The interaction of three different factors, i.e., time, condition and state were determined using a mixed model repeated measures MANOVA. To further elucidate the nature of identified interactions between groups, the data were analyzed by one way ANOVA. Firstly, overall statistical significance was determined for the 24-h period between the treatment groups (baseline and SF). In addition, statistical significance for 2-h bins for 24 h was assessed, followed by post-hoc Holm-Sidak analyses, as needed. Similar statistical approaches were used to compare delta power during SWS, number of wake episodes and the latency of SWS. Similar statistics were carried out on above mentioned parameters for TNF-α Ab injection and saline injection studies. For all comparisons, a p value<0.05 was considered to achieve statistical significance.

## Results

All mice including the TNFR KO mice recovered from surgery without any signs of infection. These mice did not behave differently from WT mice in their usual behaviors, such as eating, drinking, motor activity, and sleep postures. Also no atypical features were observed in the EEG and EMG recordings and EEG-FFT analysis from either WT mice or TNFR KO mice. WT mice subjected to TNF-α Ab also showed no deficits in their overall behaviors.

### Group 1

**Figure 1:**
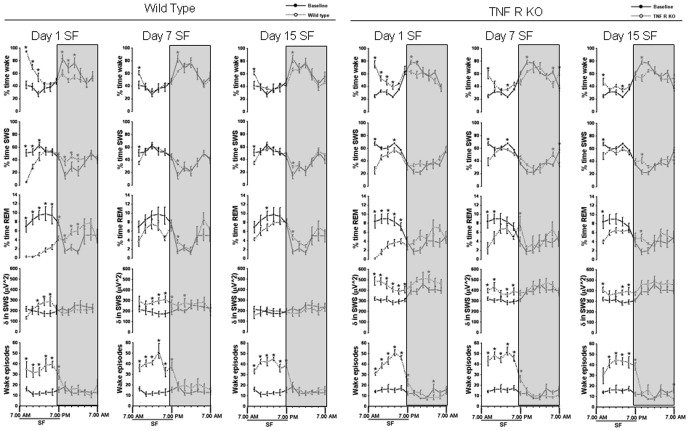
Sleep-wakefulness, EEG delta power in SWS and wake episodes (2 hr bins) in wild type and TNF R KO mice. Comparison of sleep parameters in WT and TNFR KO mice at baseline (solid line represents baseline values in the respective group), day 1, day 7 and day 15 of SF (dashed line represents SF values in the respective group). Shaded area represent dark period. The black line indicates SF period (7.00 am to 7.00 pm). SF, sleep fragmentation. p<0.05. * = comparison between baseline and SF during respective post-fragmented days.

**Figure 2:**
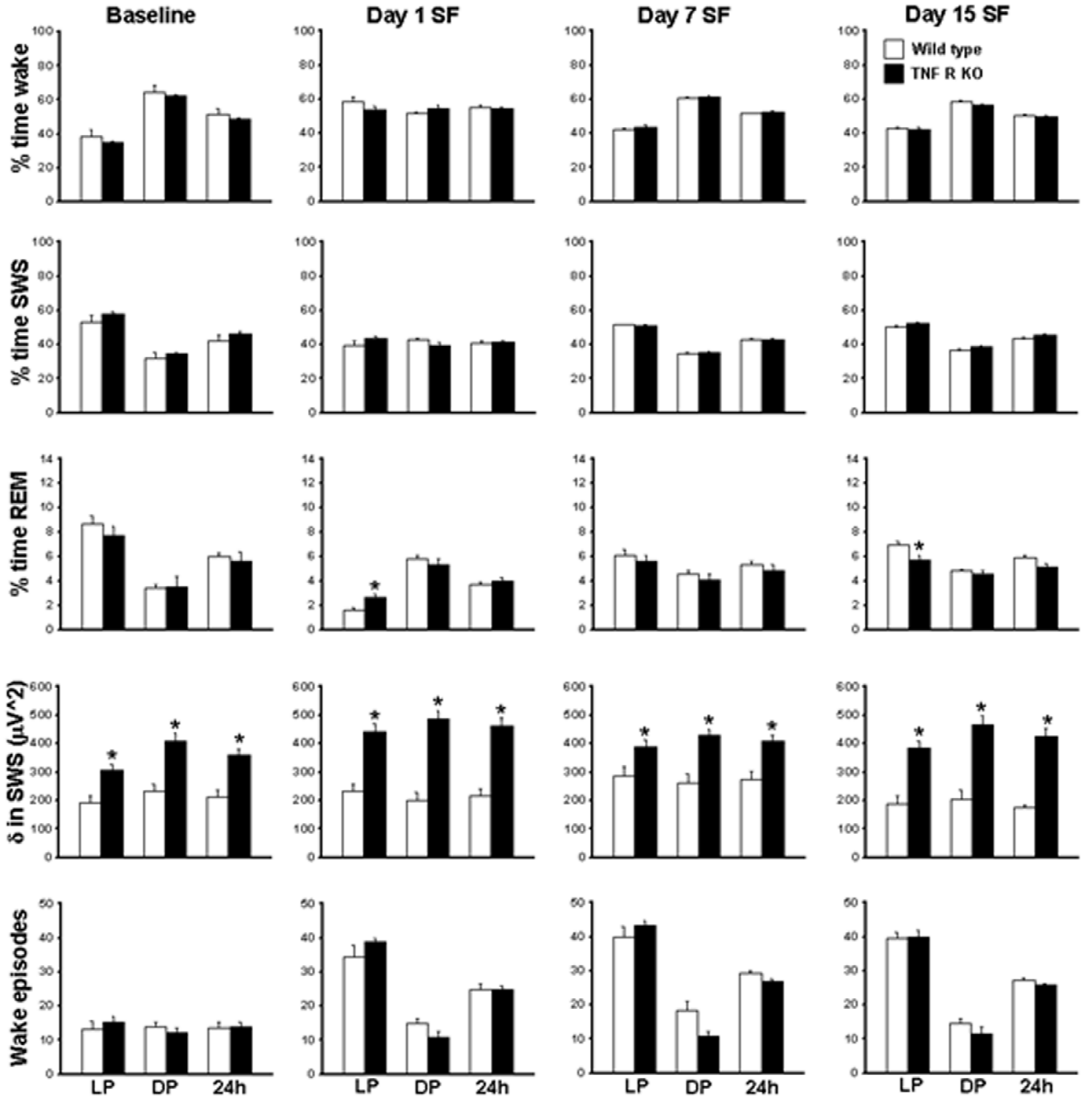
Sleep-wakefulness, EEG delta power in SWS and wake episodes (light period, dark period and total (24 h) in wild type and TNF R KO mice. Comparison of sleep parameters in WT and TNFR KO mice during light (LP), dark (DP) and 24 hours (24 h) period at baseline, day 1, day 7 and day 15 of SF in WT (open bars) and TNF R KO mice (filled bars). LP, light period; DP, dark period. *p<0.05. * = comparison between baseline and SF during respective post-fragmented days.

Baseline values for wake, SWS and REM sleep in TNFR KO mice showed no significant differences when compared to WT mice (Fig. 1 and 2; Table 1). Mice subjected to SF during day 1, spent more time in the wake state during the initial hours of SF, but subsequently manifested the same duration of wake as baseline values during the last 6 h of SF. Following chronic SF, the animals acclimatized with the SF procedure quicker, and after the first 2 h, their sleep patterns were comparable to baseline values.

**Table 1:** The percentage time spent in wake, slow wave sleep (SWS) and rapid eye movement (REM) sleep for C57BL/6J mice at baseline, day1, 7 and 15 of SF.

		Baseline	SF day 1	Baseline vs Day 1	SF day 7	Baseline vs Day 7	SF day 15	Baseline vs Day 15	
State	Time of day	Percentage time spent	Percentage time spent	Significance	Percentage time spent	Significance	Percentage time spent	Significance	Overall significance
**Wake**	7:00 AM–9.00 AM	41.78±6.15	94.56±0.78	q = 14.31, p<0.001	61.18±3.57	q = 5.26, p<0.01	61.23±2.70	q = 5.27, p<0.1	F = 35.31, p<0.001
	9:00 AM–11.00 AM	39.0±5.67	70.29±3.83	q = 11.31, p<0.001	36.9±2.16		39.85±2.74		F = 33.08, p<0.001
	11:00 AM–1.00 PM	27.4±4.42	53.6±6.74	q = 5.62, p<0.006	31.56±4.42		37.08±2.12		F = 6.088, p<0.009
	1:00 PM–3.00 PM	36.75±6.87	42.77±3.10		35±1.23		33.1±1.08		
	3:00 PM–5.00 PM	38.41±6.87	43.73±1.67		43.23±2.04		43.34±0.93		
	5:00 PM–7.00 PM	47.76±4.78	47.49±3.23		46.18±3.52		41.37±0.78		
	7:00 PM–9.00 PM	83.58±5.84	60.42±5.23	q = 6.45, p<0.002	63.29±1.08	q = 5.65, p<0.006	66.82±2.19	q = 4.67, p<0.022	F = 8.354, p<0.009
	9:00 PM–11.00 PM	68.43±7.53	48.92±3.24	q = 4.43, p<0.031	72.91±1.99		65.26±1.74		F = 5.649, p<0.01
	11:00 PM–1.00 AM	76.75±7.77	54.183.68	q = 5.29, p<0.009	76.66±3.72		76.16±0.95		F = 6.882, p<0.009
	1:00 AM–3.00 AM	59.16±8.78	49.74±3.79		59.83±2.12		55.1±2.05		
	3:00 AM–5.00 AM	44.25±5.44	44.95±7.62		40.60±2.92		39.95±6.52		
	5:00 AM–7.00 AM	55.35±7.38	51.92±3.69		50.91±2.46		46.28±1.71		
**SWS**	7:00 AM–9.00 AM	51.25±5.42	5.18±0.79	q = 13.93, p<0.001	35.08±2.7	q = 4.89, p<0.017	34.4±72.7	q = 5.07, p<0.013	F = 33.70, p<0.001
	9:00 AM–11.00 AM	52.48±5.46	29.46±3.80	q = 7.84, p<0.001	56.56±1.52		54.11±3.16		F = 18.34, p<0.001
	11:00 AM–1.00 PM	63.08±4.13	45.51±6.60	q = 4.09, p<0.040	60.8±3.5		55.97±2.52		F = 3.3, p<0.040
	1:00 PM–3.00 PM	53.43±6.28	55.48±3.32		57.93±1.06		58.96±0.85		
	3:00 PM–5.00 PM	52.13±5.61	53.84±1.97		52.31±1.97		48.54±0.82		
	5:00 PM–7.00 PM	44.45±4.35	48.148±3.08		46.63±2.84		50.25±0.51		
	7:00 PM–9.00 PM	14.91±5.68	35.68±5.05	q = 5.98, p<0.004	33.0±0.80	q = 5.20, p<0.011	28.4±1.92		F = 7.06, p<0.003
	9:00 PM–11.00 PM	29.26±7.16	45.36±2.87	q = 3.93, p<0.49	24.5±2.12		31.18±1.6		F = 4.79, p<0.015
	11:00 PM–1.00 AM	21.95±7.42	39.78±2.98	q = 4.59, p<0.025	21.38±3.37		21.04±0.87		F = 5.57, p<0.009
	1:00 AM–3.00 AM	35.83±6.89	42.715±2.96		35.98±2.0		40.02±1.76		
	3:00 AM–5.00 AM	50.53±4.69	47.28±6.64		50.58±2.17		53.04±5.3		
	5:00 AM–7.00 AM	39.61±6.09	44.34±3.61		42.71±2.41		47.71±1.47		
**REM**	7:00 AM–9.00 AM	6.96±1.29	0.27±0.17	q = 8.29, p<0.001	3.73±1.13		4.295±0.32		F = 11.617, p<0.001
	9:00 AM–11.00 AM	8.5±1.17	0.24±0.13	q = 9.87, p<0.001	6.55±1.0		6.04±0.92		F = 18.08, p<0.001
	11:00 AM–1.00 PM	9.51±1.0	0.82±0.37	q = 11.22, p<0.001	7.65±1.12		6.94±0.74		F = 23.66, p<0.001
	1:00 PM–3.00 PM	9.81±1.52	1.74±0.45	q = 8.02, p<0.001	7.06±0.89		7.92±0.45		F = 11.82, p<0.001
	3:00 PM–5.00 PM	9.45±1.90	2.41±0.55	q = 6.53, p<0.002	4.45±0.51	q = 4.64, p<0.023	8.11±0.36		F = 9.07, p<0.001
	5:00 PM–7.00 PM	7.8±0.68	4.36±0.54	q = 4.60, p<0.025	7.2±1.17		8.37±0.45		F = 5.67, p<0.008
	7:00 PM–9.00 PM	1.48±0.46	3.89±0.49	q = 5.62, p<0.006	3.70±0.45	q = 5.19, p<0.011	4.77±0.57	q = 7.68, p<0.001	F = 10.68, p<0.001
	9:00 PM–11.00 PM	2.33±0.66	5.72±0.68	q = 6.18, p<0.003	2.56±0.32		3.55±0.28		F = 7.95, p<0.002
	11:00 PM–1.00 AM	1.3±0.43	6.04±0.84	q = 8.36, p<0.001	1.95±0.49		2.79±0.29		F = 13.79, p<0.001
	1:00 AM–3.00 AM	5.03±2.0	7.54±1.10		4.2±0.42		4.87±0.36		
	3:00 AM–5.00 AM	5.2±1.30	7.76±10.1		8.80±1.30		7.0±1.35		
	5:00 AM–7.00 AM	5.03±1.37	3.74±0.47		6.36±0.61		6.0±0.36		

Data are expressed mean±SEM.

Multivariate analysis showed that behavioral state for WT mice was found to vary with time, state and condition, as reflected in a significant two-way interaction of time×state (F_(22,100)_ = 5.936, p<0.0001) and condition×state (F_(72, 318)_ = 3.444, p<0.0001). Furthermore, the three-way interaction of time×condition×state showed that the experimental manipulations did have an influence on state and across 24 h recordings (F_(66,324)_ = 3.739, p<0.0001). Behavioral state for TNFR KO was also found to vary with time, state and condition, as reflected in a significant two-way interaction of time×state (F_(22,100)_ = 4.627, p<0.0001) and condition×state (F_(72,318)_ = 2.650, p<0.0001). The three-way interaction of time×condition×state showed that the experimental manipulations did have an influence on state and across 24 h recordings (F_(66,324)_ = 2.669, p<0.0001).

### Sleep-wake comparison in WT and TNFR KO mice

On day 1 of SF both WT (58.75±2.56; F = 16.252, p<0.001) and TNFR KO (53.91±1.56; F = 53.208, p<0.001) showed a significant increase in percent time wake (Fig. 1 and 2, Table 1). The increased wake seen on day 1 SF was ameliorated by day 7 (42.34±0.39) and day 15 (42.66±0.85) in WT (Fig. 1 and 2, Table 1 and 2). TNFR KO mice also showed similar trend in wake (day 7, 43.56±1.07 and day 15, 42.24±0.99). During 12 h DP the WT mice were awake for 64.59±3.66 and TNFR KO were awake for 62.13±0.81% of the time. On day 1 of SF, both WT (51.69±0.88; F = 7.28, p<0.003) and TNFR KO (54.64±2.14; F = 8.993, p<0.001) showed a significant decrease in percent time wake (Fig. 1 and 2, Table 1 and 2). The decreased wake seen on day 1 SF during the DP returned back to baseline values by day 7 (60.70±0.48) and day 15 (58.27±0.78) in WT (Fig. 1 and 2, Table 2). Total time (24 h) showed no significant difference in wake in both WT and TNFR KO mice (Fig. 2).

**Table 2:** The percentage time spent in wake, slow wave sleep (SWS) and rapid eye movement (REM) sleep for TNF R KO mice at baseline, day1, 7 and 15 of SF.

		Baseline	SF day 1	Baseline vs Day 1	SF day 7	Baseline vs Day 7	SF day 15	Baseline vs Day 15	
State	Time of day	Percentage time spent	Percentage time spent	Significance	Percentage time spent	Significance	Percentage time spent	Significance	Overall significance
**Wake**	7:00 AM–9.00 AM	24.61±2.4	74.71±5.83	q = 11.29, p<0.001	58.73±6.66	q = 7.68, p<0.001	47.75±5.39	q = 5.21, p<0.11	F = 22.48, p<0.011
	9:00 AM–11.00 AM	32.48±2.25	52.58±4.29	q = 5.21, p<0.011	42.85±5.40		34.98±1.97		F = 5.51, p<0.009
	11:00 AM–1.00 PM	30.73±4.13	46.75±4.48	q = 4.27, p<0.039	33.82±2.92		41.08±2.37		F = 3.709, p<0.039
	1:00 PM–3.00 PM	23.62±0.84	38.95±3.61	q = 6.39, p<0.002	35.33±1.98	q = 4.685, p<0.022	34.15±2.75	q = 4.39, p<0.033	F = 7.54, p<0.002
	3:00 PM–5.00 PM	35.53±1.20	44.68±0.62		43.92±2.94		39.59±3.62		
	5:00 PM–7.00 PM	61.28±2.70	65.81±6.35		46.73±4.86		55.89±6.04		
	7:00 PM–9.00 PM	78.5±3.53	62.8±2.87	q = 4.97, p<0.015	63.7±1.48	q = 4.68, p<0.002	53.49±5.46	q = 7.91, p<0.001	F = 10.712, p<0.001
	9:00 PM–11.00 PM	76.9±2.60	58.45±5.30		68.82±7.05		64.88±1.96		
	11:00 PM–1.00 AM	61.45±1.57	62.58±7.51		72.23±5.23		65.67±6.02		
	1:00 AM–3.00 AM	55.82±4.75	57.31±8.93		56.08±3.58		50.98±2.52		
	3:00 AM–5.00 AM	62.80±2.70	48.77±4.28		42.88±4.83	q = 4.89, p<0.017	51.82±5.41		F = 4.21, p<0.024
	5:00 AM–7.00 AM	37.32±4.184	37.92±5.82		61.96±4.58	q = 4.38, p<0.033	52.5±5.57		F = 4.53, p<0.019
**SWS**	7:00 AM–9.00 AM	67.42±2.67	25.1±5.80	q = 10.28, p<0.001	38.8±6.12	q = 6.95.28, p<0.001	48.29±5.58	q = 4.64, p<0.023	F = 18.66, p<0.023
	9:00 AM–11.00 AM	59.44±1.99	45.82±3.99		52.33±4.81		59.02±2.24		
	11:00 AM–1.00 PM	60.52±3.16	50.03±3.93		59.46±2.29		52.37±2.61		
	1:00 PM–3.00 PM	67.94±0.8	57.35±3.61	q = 4.82, p<0.018	57.83±1.36	q = 4.60, p<0.025	59.58±2.71		F = 5.05, p<0.025
	3:00 PM–5.00 PM	57.61±0.87	51.28±0.90		51.26±2.76		53.79±3.35		
	5:00 PM–7.00 PM	35.67±2.14	31.21±5.67		45.35±3.26		39.5±5.13		
	7:00 PM–9.00 PM	22.87±3.64	33.4±2.63		33.71±1.41		41.61±5.02	q = 5..58, p<0.004	F = 5.77, p<0.004
	9:00 PM–11.00 PM	21.70±2.77	36.18±4.3		27.73±5.39		31.51±1.89		
	11:00 PM–1.00 AM	34.01±1.7	32.86±6.62		25.28±4.58		31.37±5.13		
	1:00 AM–3.00 AM	38.14±4.02	35.48±7.45		38.91±3.18		42.99±2.29		
	3:00 AM–5.00 AM	33.46±2.34	44.28±3.8		50.33±3.65	q = 4.95, p<0.015	42.80±4.24		F = 4.197, p<0.015
	5:00 AM–7.00 AM	57.67±4.21	57.85±5.31		33.88±3.45	q = 4.78, p<0.019	41.76±4.87		F = 5.60, p<0.018
**REM**	7:00 AM–9.00 AM	8.43±1.5	0.18±0.16	q = 8.76, p<0.001	2.46±1.32	q = 6.34, p<0.002	3.95±0.43	q = 4.75, p<0.020	F = 13.06, p<0.02
	9:00 AM–11.00 AM	8.98±1.05	1.6±0.59	q = 7.49, p<0.001	4.8±1.36	q = 4.21, p<0.042	5.99±0.62		F = 9.601, p<0.042
	11:00 AM–1.00 PM	9.05±1.3	3.22±0.76		6.73±0.98		6.54±0.58		F = 6.468, p<0.003
	1:00 PM–3.00 PM	8.43±1.8	3.7±0.73	q = 6.18, p<0.003	6.83±1.13		6.25±0.37		F = 4.531, p<0.019
	3:00 PM–5.00 PM	7.28±1.1	4.03±0.57	q = 4.64, p<0.023	4.81±0.43		6.61±0.59		F = 4.703, p<0.017
	5:00 PM–7.00 PM	3.9±1.05	2.97±0.93		7.93±1.60	q = 4.19, p<0.001	4.60±1.32		F = 5.04, p<0.011
	7:00 PM–9.00 PM	1.76±0.3	3.8±0.51		2.58±0.47		4.89±0.77	q = 5.34, p<0.009	F = 5.495, p<0.009
	9:00 PM–11.00 PM	2.04±0.3	5.37±1.11		3.45±1.77		3.6±0.19		
	11:00 PM–1.00 AM	4.2±1.23	4.55±1.0		2.48±0.73		2.95±1.02		
	1:00 AM–3.00 AM	4.08±1.23	7.2±1.58		5.01±0.60		6.025±0.84		
	3:00 AM–5.00 AM	3.68±1.0	6.95±0.78		6.78±1.56		5.36±1.19		
	5:00 AM–7.00 AM	5.03±1.22	4.23±0.67		4.15±1.28		4.71±0.83		

Data are expressed mean±SEM.

On day 1 of SF, both WT (39.60±2.51; F = 8.45, p<0.002) and TNFR KO (43.46±1.48; F = 45.353, p<0.001) showed a significant decrease in percent time SWS (Fig. 1 and 2, Table 1). The decreased SWS seen on day 1 SF returned back to baseline values by day 7 (51.55±0.35) and day 15 (50.38±0.86) in WT (Fig. 1 and 2, Table 2). TNFR KO mice also showed a similar trajectory in SWS (day 7, 50.84±1.05 and day 15, 52.09±1.13). The WT mice were in SWS for 32.01±3.51% and TNFR KO were in SWS for 34.64±0.87% of time during dark period. On day 1 of SF, only WT (42.52±0.89; F = 5.78, p<0.008) showed a significant increase in percent time SWS (Fig. 1 and 2, Table 1 and 2). TNFR KO mice, on the other hand, did not show increased SWS during DP on day1 (39.54±1.79%), day 7 (34.97±0.96%) and day 15 (38.86±0.8%) (Fig. 1 and 2). There was no change in total time (24 h) spent in SWS both in WT and TNFR KO mice (Fig. 2).

On day 1 of SF both WT (1.64±0.19, F = 52.88%, p<0.001) and TNFR KO (2.61±0.33%; F = 18.2, p<0.001]) showed a significant decrease in REM sleep (Fig. 1 and 2, Table 1 and 2). REM sleep was significantly decreased throughout the SF procedure both in WT (6.1±0.44% on day 7 and 6.9±0.33% on day 15) and in TNFR KO (5.6±0.51% on day 7 and 5.6±0.44% on day 15) (Fig. 1 and 2; see Fig. 1 for 2 hourly bins). During 12 h baseline DP the WT mice were in REM sleep for 3.4±0.35% and TNFR KO were in REM stage for 3.47±0.88% of time. On day 1 of SF both WT (5.78±0.31%; F = 14.96, p<0.001) and TNFR KO (5.35±0.4%; F = 3.48, p<0.04) showed a significant increase in percent time REM (Fig. 1 and 2, Table 1 and 2), indicating REM sleep rebound. Following chronic SF, both the WT and TNF R KO showed persistent increase in REM sleep [(WT: day 7, 4.6±0.26% and day 15, (4.83±0.13%) ; (TNF R KO: day 7, 4.07±0.44% and day 15, 4.6±0.23%)] (Fig. 1 and 2, Table 2). During the baseline 24 h period WT spent 6.03±0.24% and TNF R KO spent 5.75±0.8% percent of time in REM sleep. The WT mice showed significant decreases in total time (24 h) spent in REM on day 1 (3.71±0.24%) and day 7 (5.35±0.3%); (F = 40.58, p<0.001) (Fig. 2). There was no change in total time (24 h) spent in REM in TNF R KO.

### EEG delta power during SWS

The baseline values of absolute SWS delta power in TNFR KO mice were significantly higher during LP (305.94±19.45 µV∧2 in TNFR KO and 192.88±25.09 µV∧2 in WT mice) and DP (409.82±26.63 µV∧2 in TNFR KO and 232.41±25.71 µV∧2 in WT mice) (Fig. 1). During the DP, the TNFR KO mice showed significant increases in percent delta at 9–11pm (92.74±10.03 µV∧2 in WT and 132.05±10.92 µV∧2 in TNFR KO; p<0.02) and 1–3am (86.48±9.57 µV∧2 in WT and 127.14±8.6 µV∧2 in TNFR KO; p<0.019) (Fig. 4). Similarly, on day 7 and 15, the percentage of delta during DP was significantly increased in TNFR KO when compared to WT (Fig. 4). Although the TNFR KO exhibited significantly higher absolute delta power than the WT mice [p<0.001], (Fig. 1), the percentage change from baseline showed only a trend level increase (Fig. 4).

### Wake episodes

The number of wake episodes at baseline in WT was 13.34±2.08/h and in TNFR KO were 15.28±1.56/h. There was significant increase in wake episodes from 7 am to 7 pm during the SF procedures, indicating both WT and TNFR KO mice awakened as intentionally planned during SF implementation (Fig. 1 and 2). During SF procedures, the WT had 34.52±3.25 episodes of wake/h on day 1, 39.97±2.80/h on day 7 and 39.58±1.77/h on day 15 (F = 40.02, p<0.001). A similar trend was found in TNFR KO mice (Day 1, 38.83±0.88/h, day 7, 43.25±1.34/h and day 15, 40.0±1.9/h; F = 67.4, p<0.001). There was no difference in the number of wake episodes following cessation of SF procedures during DP both in WT and TNFR KO on all recording days (Fig. 1 and 2).

### Slow wave sleep latency

**Figure 3:**
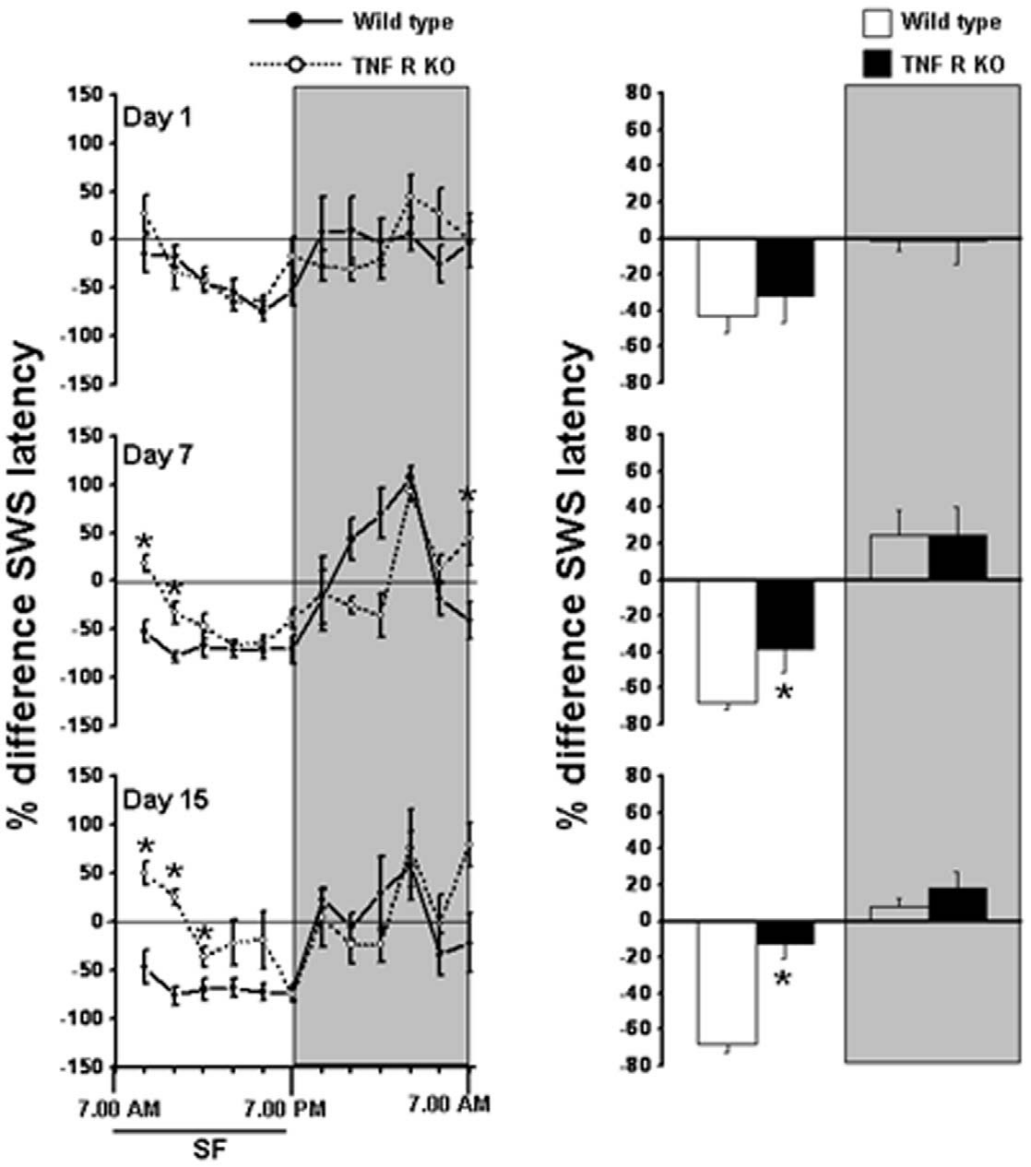
SWS sleep latency in WT and TNF R KO mice. Left panel shows percent differences in SWS latency in WT (solid line) and TNFR KO mice (dashed line) (2 hr bins) on day 1, day 7 and day 15 of SF. Right panel shows total SWS latency during light period and dark period in WT (open bars) and TNF R KO mice (filled bars). Shaded area represent dark period. The black line indicates SF period (7.00 am to 7.00 pm). SF, sleep fragmentation. p<0.05. * = comparison between WT and TNF R KO during respective post-fragmented days.

The latency to SWS was significantly reduced through out the light period during the SF procedures in WT mice only, day15>day 7>day 1, indicating progressive increase in sleep pressure. On day 1, the percent change in SWS latency during LP in WT (−43.06±9.42%) and TNFR KO mice (−32.36±4.33%) were similar. However, on day 7, WT animals had significant lower SWS latency (−68.44±8.66% in WT and −38.62±2.72% in TNFR KO, F = 10.784, p<0.008) and on day 15, the percent difference in SWS latency further reduced in WT when compared to TNFR KO (−67.92±9.78% in WT and −12.78±8.11% in TNF R KO, F = 18.823, p<0.001) (Fig. 3). After cessation of SF procedures, during DP, the latency to SWS showed a tendency to return to baseline levels (Fig. 3).

**Figure 4:**
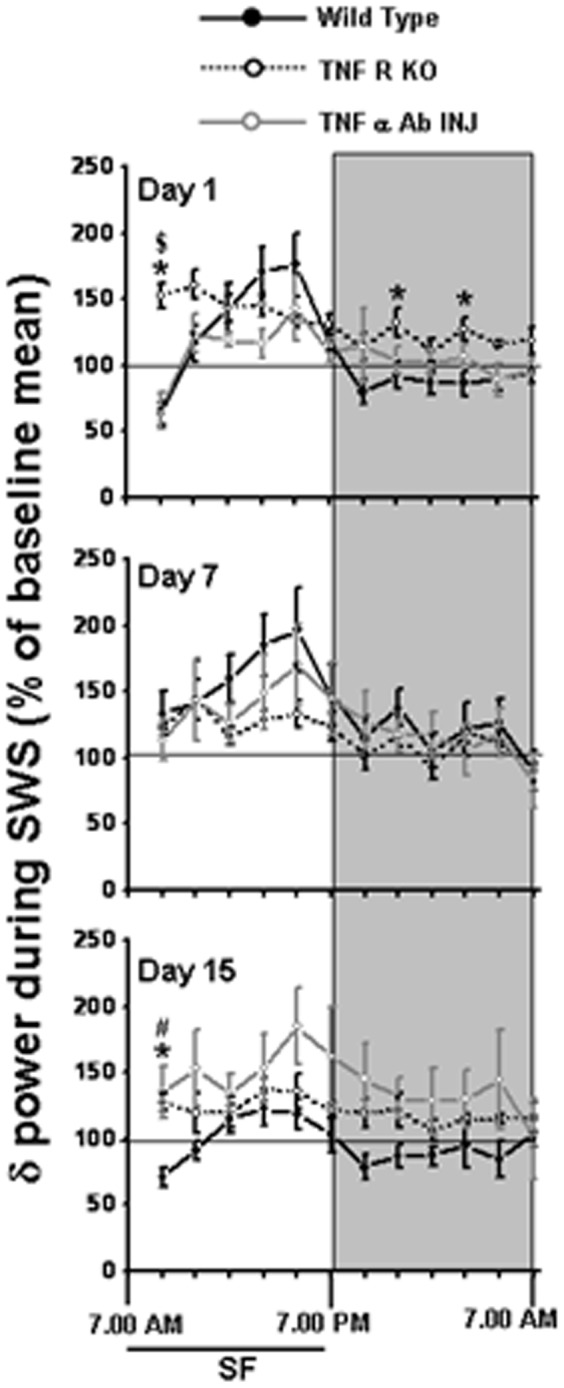
EEG delta power during SWS in WT, TNF R KO and TNF-α antibody injection mice. Comparison of normalized EEG delta power in WT (solid bar), TNF R KO (dashed line) and TNF-α antibody injection (grey mice). The black line indicates SD period (7.00 am to 7.00 pm). ). SF, sleep fragmentation. p<0.05. * = comparison between WT and TNF R KO during respective post-fragmented days. $ = comparison between WT and TNF R KO.

### Group 2

Multivariate analysis showed that behavioral state for TNF-α Ab was found to vary with time, state and condition, as reflected in a significant two-way interaction of time×state (F_(22,100)_ = 4.362, p<0.0001) and condition×state (F_(72,318)_ = 2.428, p<0.0001). Furthermore, the significant three-way interaction of time×condition×state showed that the experimental manipulations did have an influence on state and across 24 h recordings (F_(66,324)_ = 2.646, p<0.0001).

### Effect of TNF neutralizing antibody on S-W

**Figure 5:**
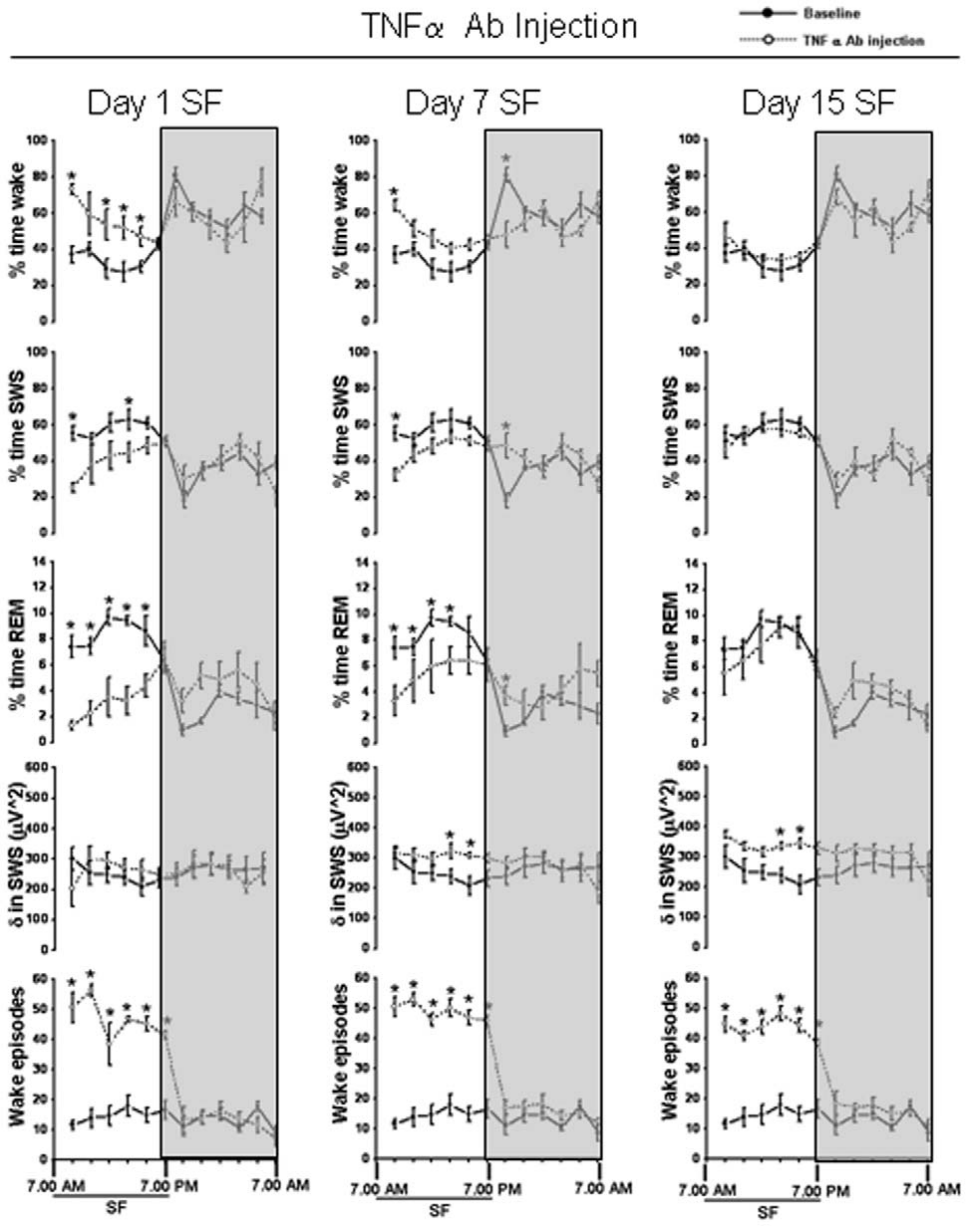
Sleep-wakefulness, EEG delta power in SWS and wake episodes (2 hr bins) in TNF-α antibody injection mice. Comparison of sleep parameters in TNF-α antibody injection mice at baseline (solid line represents baseline values in the respective group), day 1, day 7 and day 15 of SF (dashed line represents SF values in the respective group). Shaded area represent dark period. The black line indicates SF period (7.00 am to 7.00 pm). SF, sleep fragmentation. p<0.05. * = comparison between baseline and SF during respective post-fragmented days.

24-hour analyses of EEG of mice injected with saline and subjected to SF for 15 days showed comparable results to those of WT mice subjected to SF alone (data not shown). During baseline LP, mice were awake for 34.53±2.62% of time. However, the TNF-α Ab mice showed a significant increase in percent time wake (day 1, 54.94±3.71% and day 7, 48.40±1.56%; F = 13.180, p<0.001) (Fig. 5, table 3). By day 15 they returned to baseline levels (34.83±0.39%). During 12 h baseline DP the mice were awake for 62.5±2.6%t of time. There was no significant change in amount of percent wake on day 1 (58.88±3.41%), day 7 (55.11±0.75%) and day 15 (58.88±1.94%) in TNF-α Ab+SF (Fig. 5, Table 3), as opposed to saline-treated controls that exhibited similar changes to those seen in WT (as described above). The percent total time (for the 24-h period) spent in wake during baseline was 48.51±1.81% and with TNF-α Ab+SF, the total wake was significantly higher on day 1 (56.91±2.17%; p<0.012) and on day 7 (51.76±1.03%; p<0.017) and on day 15 (48.85±0.70%) the percentage wake returned to baseline values (Fig. 5, Table 3).

**Table 3:** The percentage time spent in wake, slow wave sleep (SWS) and rapid eye movement (REM) sleep for TNFa Ab mice at baseline, day1, 7 and 15 of SF.

		Baseline	SF day 1	Baseline vs Day 1	SF day 7	Baseline vs Day 7	SF day 15	Baseline vs Day 15	
State	Time of day	Percentage time spent	Percentage time spent	Significance	Percentage time spent	Significance	Percentage time spent	Significance	Overall significance
**Wake**	7:00 AM–9.00 AM	37.23±4.51	73.38±2.82	q = 7.376, p<0.001	64.26±3.24	q = 5.51, p<0.007	48.06±6.10		F = 10.98, p<0.001
	9:00 AM–11.00 AM	40.20±3.58	59.63±11.63		51.33±4.45		36.78±3.09		
	11:00 AM–1.00 PM	29.23±5.58	53.53±8.5	q = 4.109, p<0.048	46.0±5.02		34.6±1.64		F = 3.445, p<0.044
	1:00 PM–3.00 PM	27.55±5.47	52.11±6,39	q = 5.9, p<0.004	40.66±±2.81		33.6±2.77		F = 6.42, p<0.005
	3:00 PM–5.00 PM	30.53±3.30	47.03±5.32	q = 4.66, p<0.023	42.33±3.15		35.78±2.06		F = 4.20, p<0.024
	5:00 PM–7.00 PM	42.43±2.50	43.95±2.34		45.83±2.15		44.15±2.20		
	7:00 PM–9.00 PM	80.96±4.08	66.38±7.93		48.0±7.14	q = 6.90, p<0.001	68.15±4.16		F = 8.09, p<0.002
	9:00 PM–11.00 PM	62.23±0.99	60.46±5.36		55.33±4.99		55.51±9.07		
	11:00 PM–1.00 AM	57.36±4.31	52.18±6.38		63.16±3.49		62.91±3.7		
	1:00 AM–3.00 AM	51.83±4.42	43.38±5.05		46.16±4.76		43.2±5.46		
	3:00 AM–5.00 AM	64.33±6.88	53.61±9.49		50.38±2.84		51.75±2.52		
	5:00 AM–7.00 AM	58.26±4.09	77.25±7.31		67.67±4.22		71.76±5.90		
**SWS**	7:00 AM–9.00 AM	55.35±4.08	25.31±2.70	q = 7.12, p<0.001	32.43±3.40	q = 5.43, p<0.008	46.38±4.61		F = 10.30, p<0.001
	9:00 AM–11.00 AM	52.31±3.30	38.08±10.76	NS	43.8±4.28		56.73±2.04		
	11:00 AM–1.00 PM	61.05±5.37	42.95±7.69	NS	48.01±4.40		57.71±5.05		
	1:00 PM–3.00 PM	63.0±5.65	44.63±5.40	q = 4.48, p<0.029	52.88±3.16		57.31±3.40		F = 3.57, p<0.039
	3:00 PM–5.00 PM	60.83±3.33	48.56±4.48		51.25±2.62		55.08±2.10		
	5:00 PM–7.00 PM	51.11±2.37	49.46±142		48.05±2.22		50.06±2.08		
	7:00 PM–9.00 PM	18.06±3.82	30.4±7.07		48.3±6.95	q = 6.93, p<0.001	29.38±3.91		F = 8.22, p<0.008
	9:00 PM–11.00 PM	36.13±1.07	34.33±4.70		41.65±4.75		39.55±7.77		
	11:00 PM–1.00 AM	38.63±3.98	42.91±5.48		34.08±3.33		32.3±3.22		
	1:00 AM–3.00 AM	44.75±4.25	51.0±3.81		49.7±5.18		52.28±5.33		
	3:00 AM–5.00 AM	32.77±6.23	41.85±8.35		43.78±2.6		44.68±2.20		
	5:00 AM–7.00 AM	39.42±3.9	20.68±6.26		26.88±4.31		26.72±5.52		
**REM**	7:00 AM–9.00 AM	7.41±0.83	1.3±0.38	q = 6.089, p<0.003	3.3±1.18	q = 4.098, p<0.049	5.55±1.68		F = 7.018, p<0.004
	9:00 AM–11.00 AM	7.48±0.66	2.28±0.9	q = 8.54, p<0.001	4.86±0.67	q = 4.30, p<0.037	6.48±1.45		F = 13.94, p<0.001
	11:00 AM–1.00 PM	9.71±0.65	3.51±1.56	q = 6.26, p<0.003	5.98±0.84	q = 3.96, p<0.049	7.68±1.37		F = 7.051, p<0.004
	1:00 PM–3.00 PM	9.45±0.35	3.25±1.08	q = 9.18, p<0.001	6.45±0.42	q = 4.44, p<0.031	9.08±0.78		F = 18.07, p<0.001
	3:00 PM–5.00 PM	8.63±1.17	4.40±0.88	q = 5.30, p<0.009	6.41±1.07		9.13±0.74		F = 7.45, p<0.003
	5:00 PM–7.00 PM	6.45±0.86	6.58±1.22		6.11±0.53		5.78±0.52		
	7:00 PM–9.00 PM	0.97±0.39	3.21±0.92		3.7±0.30	q = 4.74, p<0.02	2.46±0.43		F = 4.29, p<0.022
	9:00 PM–11.00 PM	1.63±0.6	5.2±1.02		3.01±0.46		4.93±1.38		
	11:00 PM–1.00 AM	4.0±0.48	4.9±1.34		2.75±0.36		4.78±0.66		
	1:00 AM–3.00 AM	3.41±1.16	5.62±1.36		4.13±0.44		4.51±0.5		
	3:00 AM–5.00 AM	2.9±0.98	4.53±1.68		5.83±0.78		3.56±0.59		
	5:00 AM–7.00 AM	2.32±0.80	2.06±1.09		5.45±0.41	q = 4.6, p<0.025	1.51±0.51		F = 6.77, p<0.004

Data are expressed mean ± SEM.

The SWS were also affected by TNF-α Ab+SF. During the baseline 12 h LP, these mice spent 57.27±2.7% of time in SWS. When subjected to TNF-α Ab+SF, these mice showed significant reduction in SWS on day 1 (41.5±3.31%; p<0.003), day 7 (46.07±1.61%; p<0.03) and day 15 (53.88±1.27%; p<0.02) (Fig. 5, Table 3). During baseline DP the mice were in SWS for 34.96±2.53% of time. There was no significant change during the DP in amount of percent SWS on day 1 (36.86±3.19%), day 7 (40.73±0.66%) and day 15 (37.48±1.64%) with TNF-α Ab+SF (Fig. 5, table 3). The mice injected with neutralizing antibody and subjected to SF did not show any SWS rebound during the dark period. The baseline percent total time (24 h) spent in SWS in these mice was 46.12±2.05% and with TNF-α Ab+SF the total SWS was significantly lower only on day 1 (39.18±1.95%; p<0.04) (Fig. 5, Table 3).

The REM sleep was also affected by TNF-α Ab+SF. The mice spent 8.19±0.56% of time in REM sleep during baseline light period. When subjected to TNF-α Ab+SF, these mice showed significant reduction in REM during LP on day 1 (3.55±0.49%; p<0.001) and day 7 (5.52±0.32%; p<0.004) (Fig. 5, Table 3). However, by day 15 (7.28±2.15%) the percentage of REM sleep had returned to baseline levels. During the baseline DP, the mice were in REM sleep for 2.54±0.34% of time. Dark period REM sleep was increased significantly on day 1 (4.25±0.58%; p<0.03) and day 7 (4.14±0.27%, p<0.04), although there was no significant changes across 2-hr bins. REM sleep returned to baseline levels by day 15 (3.63±0.36%) (Fig. 5, Table 3).

### EEG delta power during SWS

**Figure 6:**
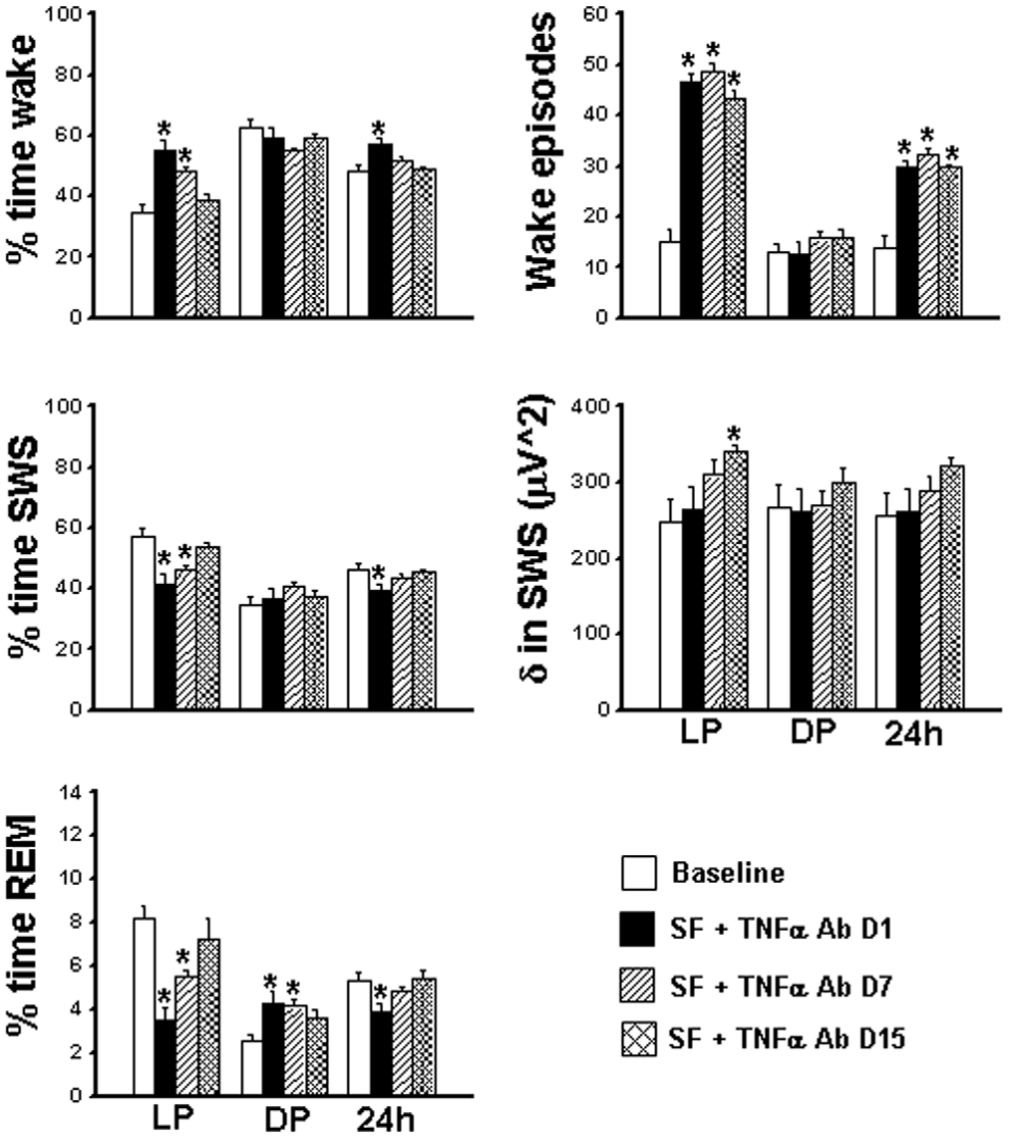
Sleep-wakefulness, EEG delta power in SWS and wake episodes (light period, dark period and total (24 h) in TNF-α antibody injection mice. Comparison of sleep parameters in TNF-α antibody injection mice during light (LP), dark (DP) and 24 hours (24 h) period at baseline (open bar), day 1 (filled bar), day 7 (hatched bar) and day 15 (double hatched bar) following SF LP, light period; DP, dark period. *p<0.05. * = comparison between baseline and SF during respective post-fragmented days.

The absolute delta power value at baseline in this group was 247.86±28.99 µV∧2. With TNF-α Ab+SF the increase in delta power during LP was not significant either on day 1 (263.68±30.23 µV∧2) or day 7 (309.51±8.86 µV∧2). However by day 15 (339.82±7.99 µV∧2; p>0.02) the mice had significant increase in absolute delta power (Fig. 5 and 6). There was no significant change in delta power during DP and total time of 24 hours across through out in TNF-α Ab+SF mice. The normalized delta in 1^st^ two hours of day 1 of TNF-α Ab+SF (65.69±14.28 µV∧2 ) was comparable to WT mice in group 1 (63.71±8.7 µV∧2), but was significantly higher in the TNFR KO mice (152.26±9.56 µV∧2, p<0.001). There was a trend toward increases in delta in both TNF-α Ab+SF and TNFR KO mice (Figs. 5 and 6).

### Wake episodes

EEG monitoring showed a significant increase in wake episodes from 7 am to 7 pm during the SF procedures in TNF-α Ab+SF mice, indicating successful intermittent arousals (Fig. 5). During the dark period, following cessation of SF procedures, the number of wake episodes was comparable to the baseline on all days. These results were virtually identical to WT mice in group 1.

### Slow wave sleep latency

**Figure 7:**
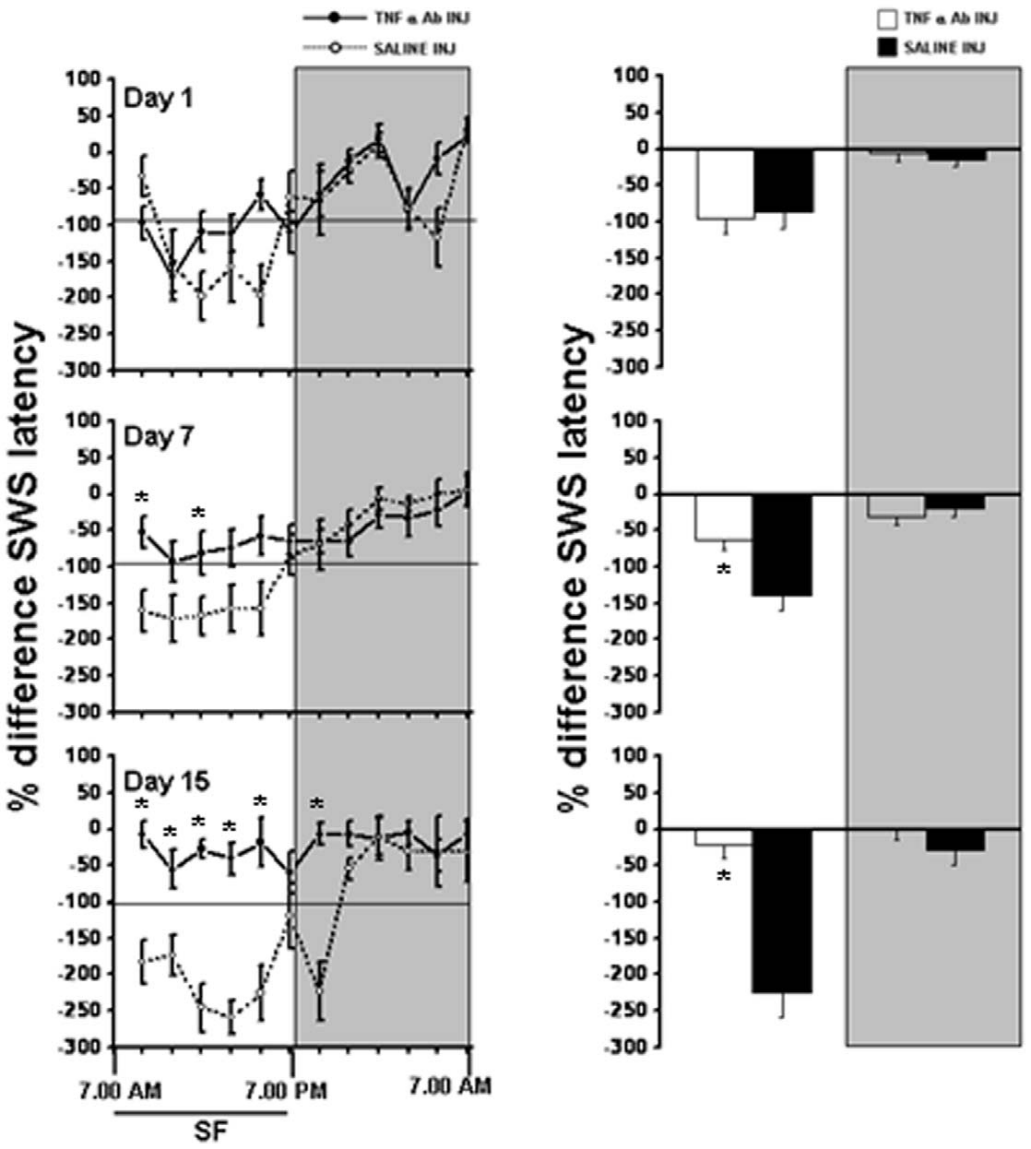
SWS sleep latency in saline and TNF-α antibody injection mice. Left panel shows percent differences in SWS latency in TNF-α antibody injection (solid line) and saline injection (dashed line) (2 hr bins) group on day 1, day 7 and day 15 of SF. Right panel shows total SWS latency during light period and dark period in TNF- α antibody injection (open bars) and saline injection (filled bars) mice. Shaded area represent dark period. The black line indicates SF period (7.00 am to 7.00 pm). SF, sleep fragmentation. p<0.05. * = comparison between TNF-α antibody injection and saline injection mice during respective post-fragmented days.

With the saline injection, the latency to SWS was significantly reduced through out the light period during the SF procedures in WT mice, day1>day 7>day 15, indicating progressive increases in sleep pressure (Fig. 7). There were no significant differences between saline (−86.857±21.45%) and TNF-α Ab+SF (−96.53±18.9%)-treated mice on day 1 during the light period. However, on day 7 the saline mice (−138.81±21.64%) showed a significant reduction in percent difference in SWS latency compared to TNF-α Ab+SF mice (−63.65±14.12%; p<0.016). On day 15, the percent difference in SWS latency was further reduced in saline mice (−223.84±35.09%) as compared to TNF-α Ab+SF (−21.39±18.19%, p<0.001) (Fig. 7), indicating that TNF-α Ab injection prevented SF-associated increases in sleep propensity. During DP, immediately following cessation of SF procedure, the latency to SWS showed a tendency to return to baseline levels (Fig. 7).

## Discussion

This study confirms that SF procedures mimicking the sleep disruption that characterizes multiple disorders in humans elicit changes in sleep propensity in the absence of major alteration in sleep duration. Furthermore, we show that TNFR KO mice as well as WT mice treated with TNF-α neutralizing antibody do not exhibit rebounds in NREM and REM sleep as compared to WT mice subjected to SF. Taken together, current experiments illustrate the temporal trajectory of sleep architecture in the context of persistent SF, and further reinforce the concept that TNF-α -mediated pathways are a critical component of SF-induced excessive sleepiness. Thus, neutralization of TNF-α biological activity may provide viable future therapeutic approaches to the excessive somnolence that characteristically accompanies the presence of frequent diseases such as OSA.

### Baseline S-W

The amount of SWS and REM sleep in the present study was comparable in WT and TNFR KO mice. These results are in agreement with a previous study [56], in which WT, ligand KO, TNFR1 KO and TNFR2 KO mice did not show any differences in their sleep architecture and patterns. However, in studies on TNFR1 KO mice by Fang *et al* [19], and in double TNFR KO by Kapas *et al* [57], KO mice slept less than WT. The reason for such discrepancies are unclear, but could be related to differences in environmental temperatures. Indeed, ambient temperature affects duration of both SWS and REM sleep [58] and TNF-α is involved in thermoregulation [59].

TNF-α signaling involves multiple adaptor proteins for both the receptors and is a complex process [60]. Since in the current study, TNFR KO mice slept similar to WT mice, it is possible that in the congenital absence of both of the TNF receptors (p55 and p75), other endogenous regulators of sleep may be playing compensatory roles to preserve sleep homeostasis. However, TNFR KO mice exhibited higher basal EEG delta power during SWS during both the light and dark periods, suggesting that TNF-α signaling may be required for normal distribution of EEG delta during SWS [57], particularly when considering that SWS and SWS delta power are two completely distinct phenomena that are controlled by different regulatory processes as suggested from pharmacological studies [61].

### Effect of SF on sleep architecture of TNFR KO

Mice exposed to SF maintained total sleep duration and globally preserved sleep state distribution and duration despite ongoing awakenings induced by the SF procedures (i.e., increased number of arousals during SF). The robust SWS rebound during the dark period seen in WT mice was absent in the TNFR KO mice. The inability of NREM and REM sleep to recuperate after 12 hours exposure to SF in TNFR KO mice suggests that these receptors play a significant role in the post-sleep disruption recovery period, particularly since SF is accompanied by elevated levels of cortical TNF-α in TNFR KO mice [6]. In another study, TNF ligand and TNFR KO mice also showed decreased REM sleep [19], [56]. Kapas et al. [57] showed typical enhanced values of SWS delta power during the dark period and an opposite trend during the light period. In this study, WT mice and TNFR KO subjected to acute (day 1) SF had significantly reduced REM sleep. However, only WT mice showed marked rebound in REM sleep during the dark period. The absence of TNF-α receptors appears to reduce the probability of initiating REM sleep, possibly by altering serotonin (5-HT) levels [56]. Indeed, TNF-α is known to increase 5-HT uptake in the brain by modulating the 5-HT transporter system [62]. Mice deficient in TNF-α gene showed enhanced levels of 5-HT in brain regions such as the medulla oblongata that have been associated with REM sleep regulation [63].

The SF procedure in WT mice enhanced SWS delta power on days 1 and 7, a decline towards baseline levels by day 15 of SF. As mentioned, TNFR KO mice had higher resting delta frequency power when compared to WT. Similar to WT, enhanced SWS delta frequency power as seen during day 1 of SF returned back to baseline levels by day 15 of SF. Both TNF ligand KO and TNFR2 knockout mice will show increases in delta power when subjected to SD [56], and current findings extend such observations to the application of a SF paradigm aimed at mimicking highly prevalent disease conditions in humans, such as OSA. The increased slow wave activity may be attributable to an exaggerated excitatory input to cortex due to the disturbance in sleep [56]. Although the total sleep time was not affected significantly by the SF procedure employed in the present study, increased SWS delta power emerged. Increased delta power during SWS is currently viewed as an indicator of increased sleep pressure [64], and is consistently documented after SD exposures. Our findings suggest that in the absence of TNF-α receptors, other homeostatic responses may take over to maintain physiological sleep by compensating the inherent sleep deficits with deeper sleep intensity. Furthermore, our findings suggest that regulation of SWS is independent from the regulation of EEG slow wave activity that is characteristically expressed as delta frequency power. Indeed, SWS latency in WT mice was incrementally reduced on days 1, 7 and 15, indicating higher sleep propensity, even when the total amount of SWS remained unaffected. On the other hand, TNFR KO mice showed no decreases in SWS latency following 15 days of SF. These findings are supportive of the concept that the increases in sleepiness induced by chronic SF are due at least in part by initiation of inflammatory signaling pathways via TNF- α. It is also worth noting however, that EEG delta power may be regulated independently of sleep duration [65].

### TNF-α neutralizing antibody injection

WT mice subjected to 15 days of TNF-α neutralizing Ab during the start of SF did not show sleep rebound during the recovery period unlike the mice injected with saline and WT mice in group 1. These results further confirm the putative assumption that endogenous TNF-α is involved in sleep regulation. Injection of either TNF-α neutralizing antibody or TNF soluble receptor inhibits endogenous TNF-α activity and hence inhibits spontaneous SWS [32], [33] and attenuates sleep rebound after sleep deprivation [28]. The attenuation of sleep rebound on day 1 in the present study was accompanied by relatively intact SWS delta frequency power during the SF procedure and immediately subsequent recovery period. TNF- α is reported to increase EEG delta power when injected [28] or applied unilaterally on cerebral cortex [30]. Therefore, the unaffected EEG delta frequency power in the present study could be due to neutralization of endogenous TNF-α, which then would abrogate the excitatory inputs that enhance delta power due to sleep disruption. Accordingly, mice injected with TNF-α neutralizing antibody had lesser sleep propensity (longer sleep latency) compared to mice injected with saline when exposed to SF. Systemic cytokines exert effects in the CNS both via direct and indirect pathways. TNF-α enters the brain through relatively permeable areas in the blood–brain barrier (BBB) [66]. In the current experimental model and in other in vivo models [67], it is possible that transient changes in BBB permeability, either caused by the systemic inflammatory response, or by other factors including the antecedent anesthesia [68], could enable direct access to the brain. Further, pre-treatment of mice with TNF-α neutralizing antibody completely protected the mice from lethal shock. [69].

In summary, TNF-α is well known for its roles in host-defense, sleep-wake regulation, and the pathogenesis of various diseases. A number of biological processes are activated by TNF-α, including sleep [40]. Higher levels of TNF-α levels are responsible for sleepiness in patients with sleep apnea, a condition that is characterized by prominent SF. Here we show that SF is indeed associated with alteration in sleep architecture and sleep propensity that appear to be modulated by TNF-α pathways. The findings from the present study suggest that blocking or neutralizing TNF-α can be helpful in ameliorating the excessive sleepiness associated with disease conditions in which SF is an integral component.
